# Supplementary figures and images for: Gut-derived peptide hormone receptor expression in the developing mouse hypothalamus
Source: PLoS One. 2023 Aug 17;18(8):e0290043. doi: 10.1371/journal.pone.0290043 (PMC10434938; doi:10.1371/journal.pone.0290043)

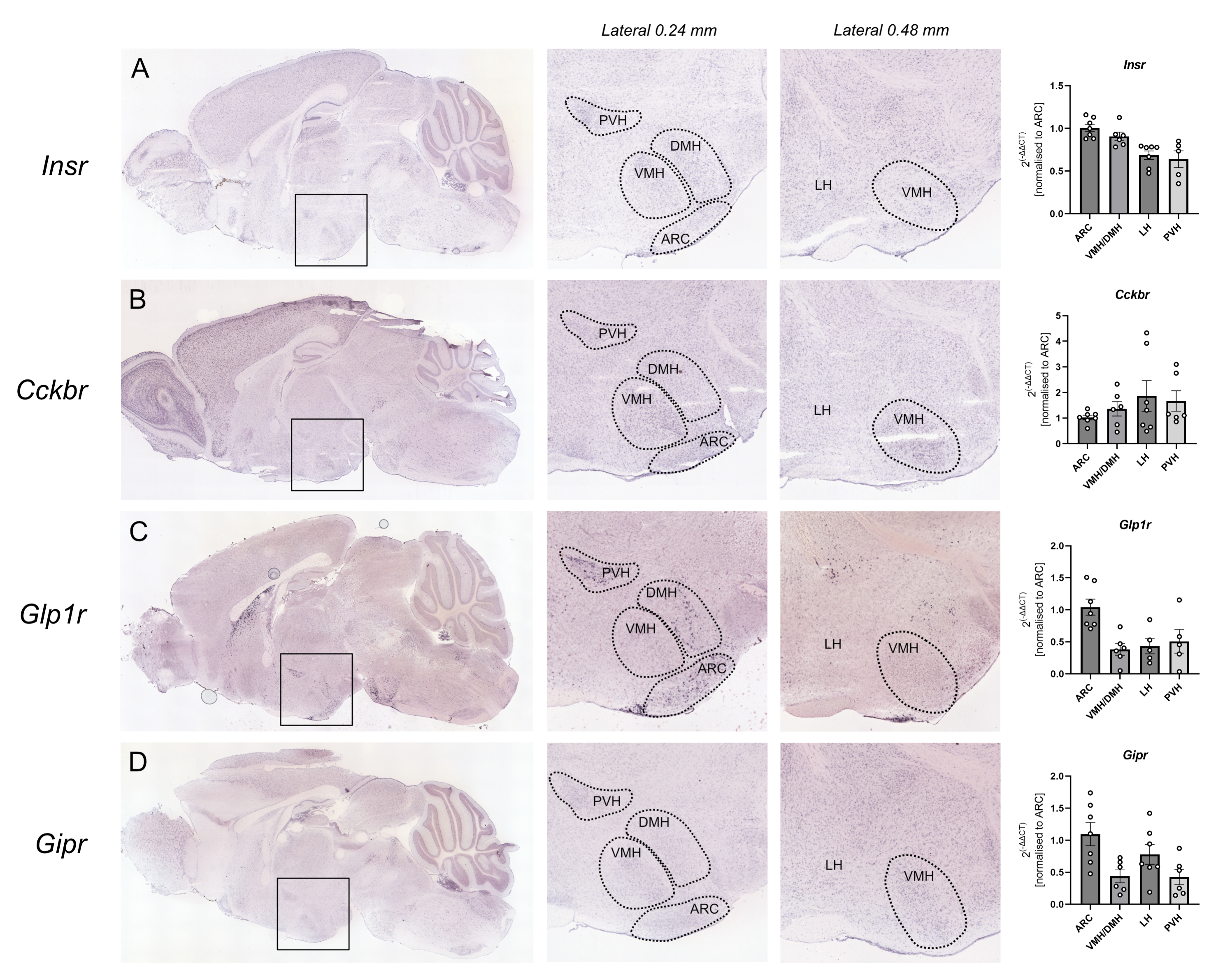

Supplement: S1 Fig — In situ hybridization images from the Allen Mouse Brain Atlas (http://mouse.brain-map.org/) corresponding to Insr (A), Cckbr (B), Glp1r (C) and Gipr (D) transcripts expression across the adult hypothalamus were aligned with data obtained from adult female mice hypothalamic subnuclei dissection, including the arcuate nucleus (ARC), the ventromedial/dorsomedial nucleus of the hypothalamus (VMH/DMH), the paraventricular nucleus of the hypothalamus (PVH) and the lateral nucleus of the hypothalamus (LH). Expression of the Insr (mouse.brain-map.org/experiment/show/69735484), Cckbr (mouse.brain-map.org/experiment/show/69236993), Glp1r (mouse.brain-map.org/experiment/show/73606497) and Gipr (mouse.brain-map.org/experiment/show/70295936) in the adult mouse brain. Open circles represent individual data points. Data is plotted as Mean ± SEM. (TIF) [file pone.0290043.s001.tif]
