# Supplementary material for: Gut-derived peptide hormone receptor expression in the developing mouse hypothalamus
Source: PLoS One. 2023 Aug 17;18(8):e0290043. doi: 10.1371/journal.pone.0290043 (PMC10434938; doi:10.1371/journal.pone.0290043)
Supplement: S1 Raw images — (PDF) [file pone.0290043.s004.pdf]

<u>INSR</u>

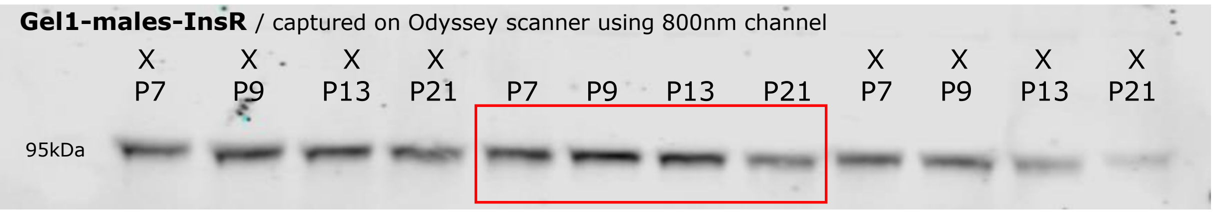

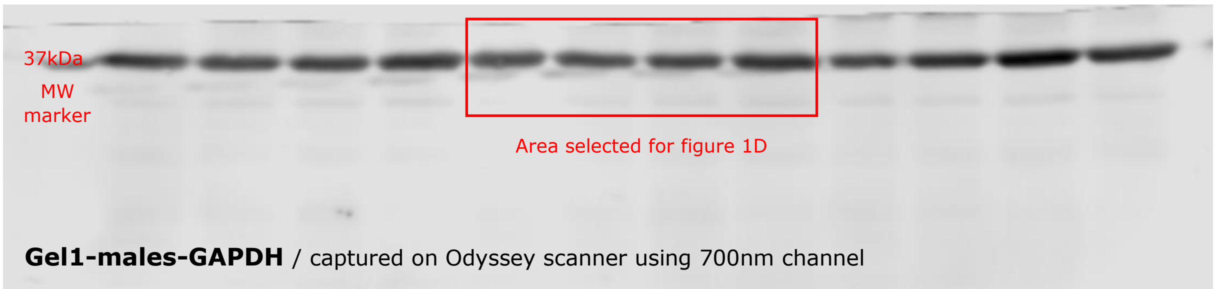

**Gel 1**	P7	P9	P13	P21	P7	P9	P13	P21	P7	P9	P13	P21
**GAPDH signal**	1300000	1110000	1160000	1420000	1160000	1140000	1110000	1460000	1030000	1370000	1770000	1500000
**InsR signal**	212000	245000	209000	193000	218000	250000	228000	157000	186000	187000	109000	49000
**InsR/GAPDH**	0,1631	0,2207	0,1802	0,1359	0,1879	0,2193	0,2054	0,1075	0,1806	0,1365	0,0616	0,0327

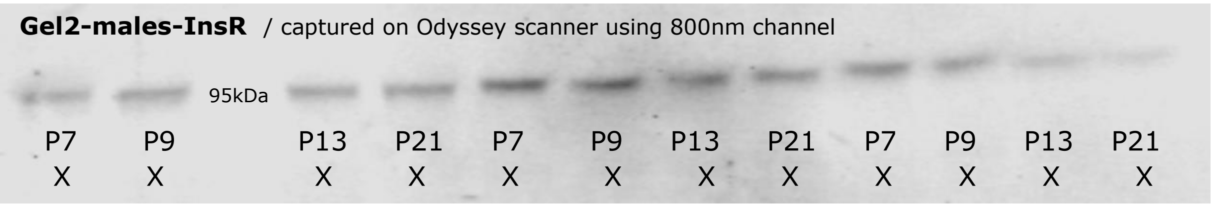

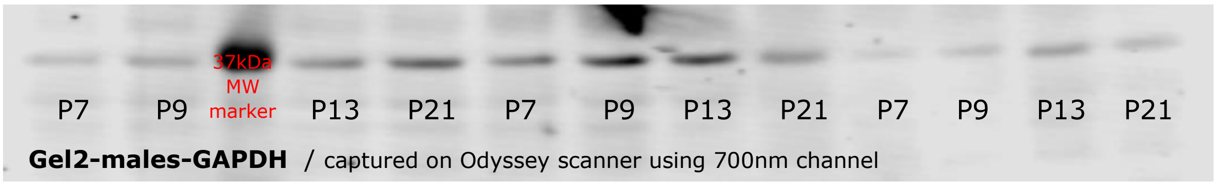

**Gel 2**	P7	P9	P13	P21	P7	P9	P13	P21	P7	P9	P13	P21
**GAPDH signal**	27900	42100	62100	98100	26300	94600	83000	57500	16200	22800	46000	33100
**InsR signal**	13600	15600	14600	15400	20700	19900	16400	15700	15100	12300	7720	4740
**InsR/GAPDH**	0,4875	0,3705	0,2351	0,1570	0,7871	0,2104	0,1976	0,2730	0,9321	0,5395	0,1678	0,1432

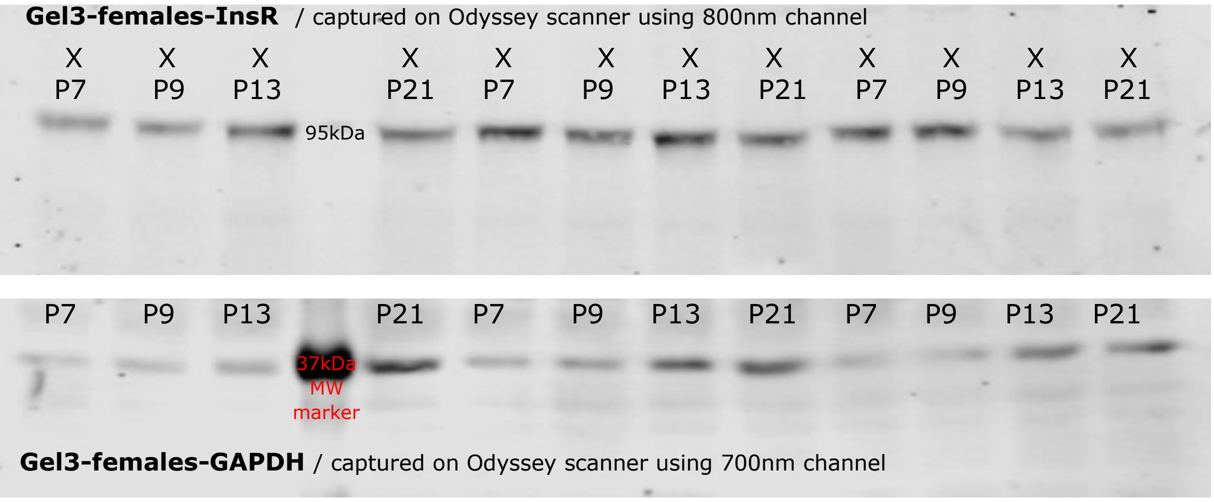

Gel 3	P7	P9	P13	P21	P7	P9	P13	P21	P7	P9	P13	P21
**GAPDH signal**	45700	52700	71500	216000	57400	69400	138000	158000	40000	46400	112000	118000
**InsR signal**	41600	37500	61100	55100	72300	58300	74600	56000	56800	59200	41800	39700
**InsR/GAPDH**	0,9103	0,7116	0,8545	0,2551	1,2596	0,8401	0,5406	0,3544	1,4200	1,2759	0,3732	0,3364

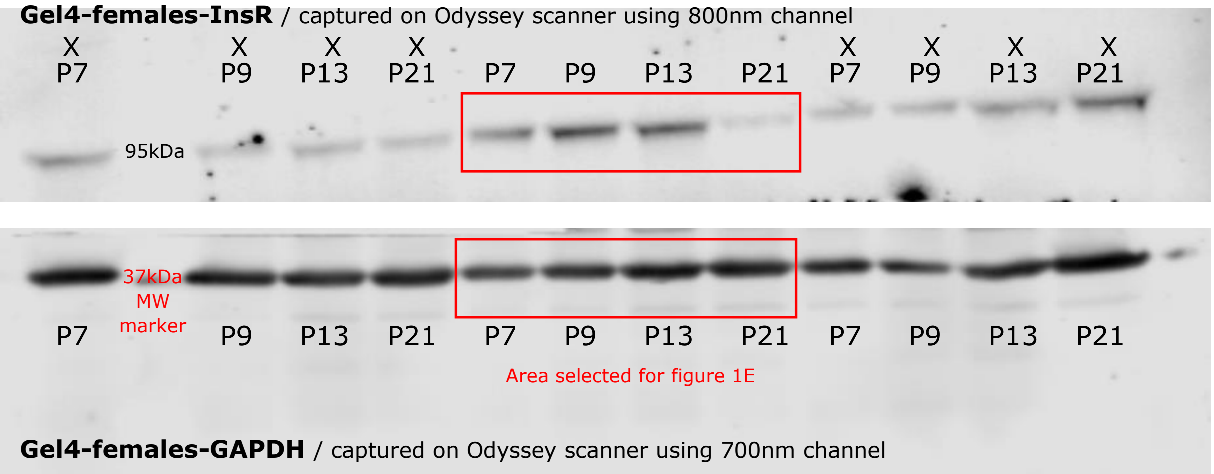

Gel 4	P7	P9	P13	P21	P7	P9	P13	P21	P7	P9	P13	P21
**GAPDH signal**	2860000	2630000	2300000	2660000	1710000	2070000	2590000	2550000	1970000	1440000	2190000	3140000
**InsR signal**	83500	41000	48700	39300	81100	136000	122000	23400	43100	50800	84800	121000
**InsR/GAPDH**	0,0292	0,0156	0,0212	0,0148	0,0474	0,0657	0,0471	0,0092	0,0219	0,0353	0,0387	0,0385

GLP1R

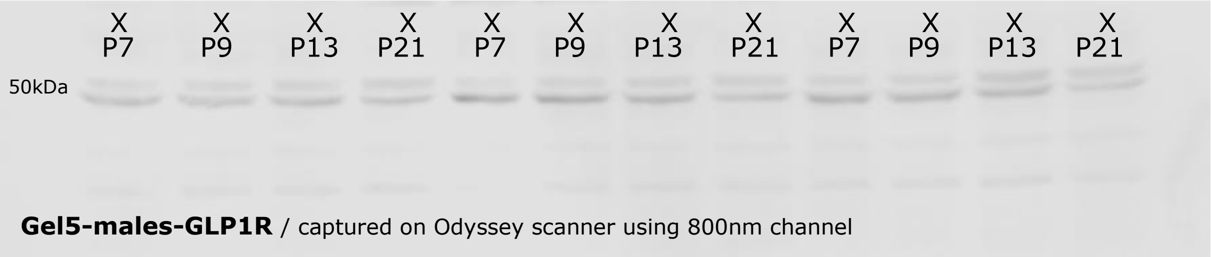

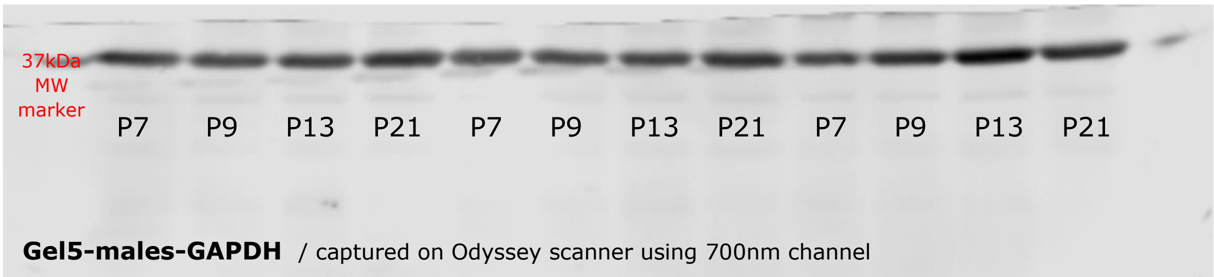

Gel 5	P7	P9	P13	P21	P7	P9	P13	P21	P7	P9	P13	P21
GAPDH signal	1300000	1110000	1160000	1420000	1160000	1140000	1110000	1460000	1030000	1370000	1770000	1500000
GLP1R signal	22200	27600	36700	57400	20600	15300	27900	55400	25000	29900	82500	51100
GLP1R/GAPDH	0,017077	0,024865	0,031638	0,040423	0,017759	0,013421	0,025135	0,037945	0,024272	0,021825	0,04661	0,034067

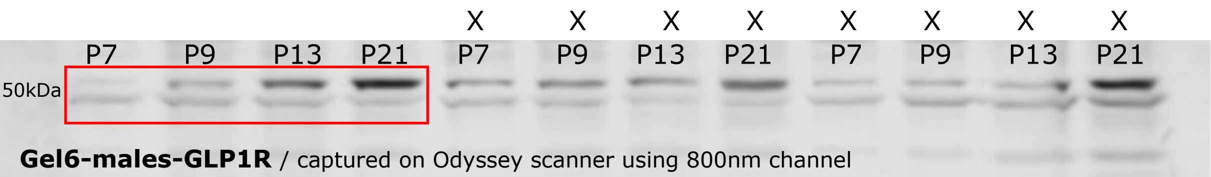

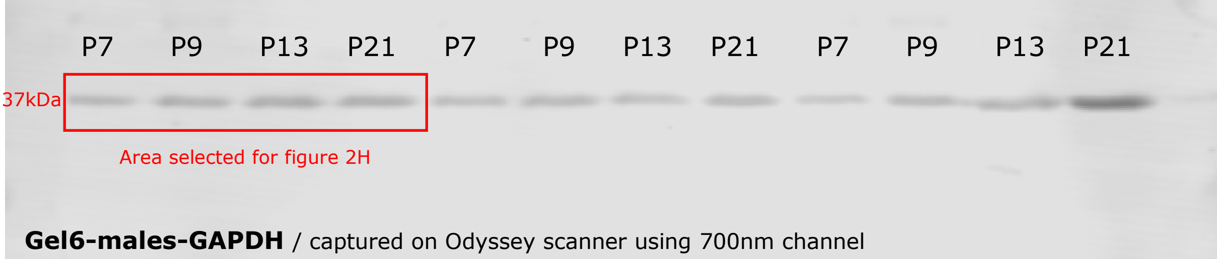

Gel 6	P7	P9	P13	P21	P7	P9	P13	P21	P7	P9	P13	P21
GAPDH signal	204000	296000	363000	377000	243000	221000	248000	261000	196000	315000	298000	781000
GLP1R signal	1630	16900	56900	103000	28300	35300	31500	65900	10100	12300	25900	87700
GLP1R/GAPDH	0,00799	0,057095	0,156749	0,27321	0,116461	0,159729	0,127016	0,25249	0,051531	0,039048	0,086913	0,112292

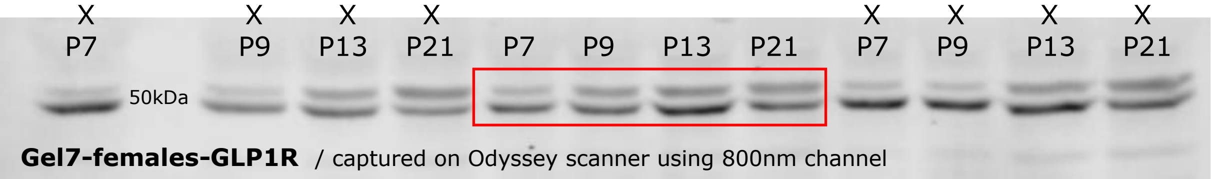

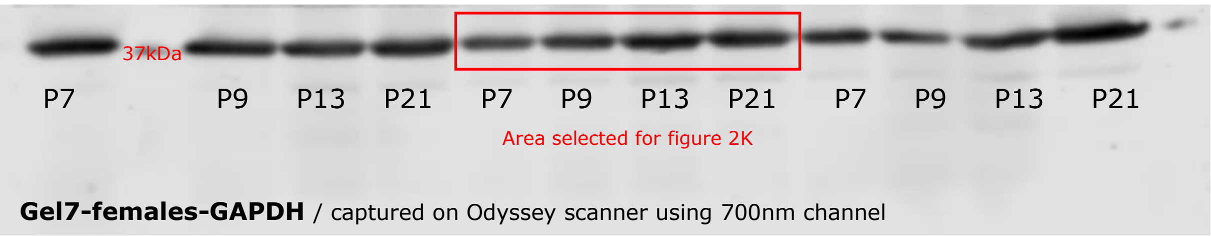

**Gel 7**	P7	P9	P13	P21	P7	P9	P13	P21	P7	P9	P13	P21
**GAPDH signal**	2860000	2630000	2300000	2660000	1710000	2070000	2590000	2550000	1970000	1440000	2190000	3140000
**GLP1R signal**	42400	23500	58300	114000	19300	66300	97400	96800	17500	13800	98200	132000
**GLP1R/GAPDH**	0,014825	0,008935	0,025348	0,042857	0,011287	0,032029	0,037606	0,037961	0,008883	0,009583	0,04484	0,042038

GIPR

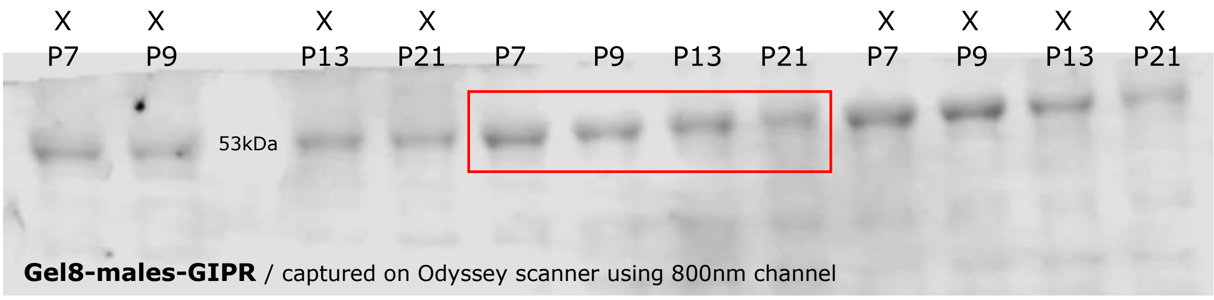

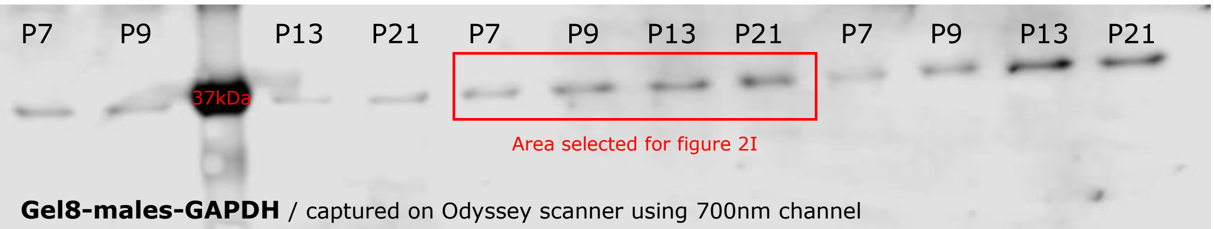

Gel 8	P7	P9	P13	P21	P7	P9	P13	P21	P7	P9	P13	P21
**GAPDH signal**	22500	33700	22900	23500	18400	20500	37500	36000	12100	27700	62600	52400
**GIPR signal**	88200	71000	70700	73500	125000	109000	87900	60100	119000	131000	89700	52600
**GIPR/GAPDH**	3,92	2,106825	3,087336	3,127659574	6,793478	5,317073	2,344	1,669444	9,834711	4,729242	1,432907	1,003817

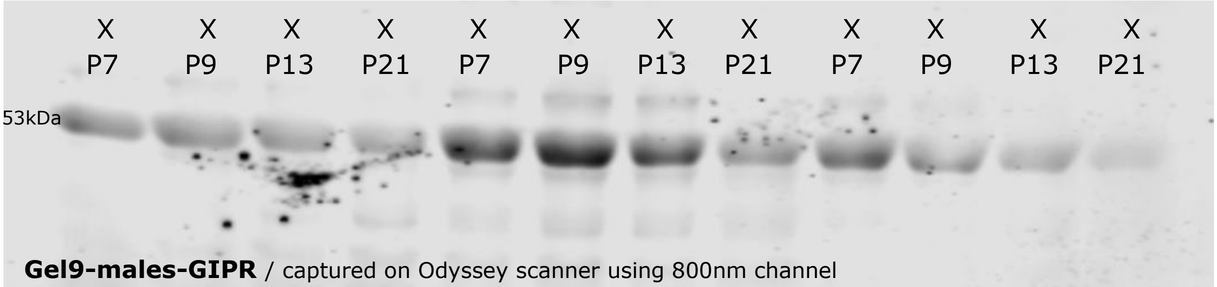

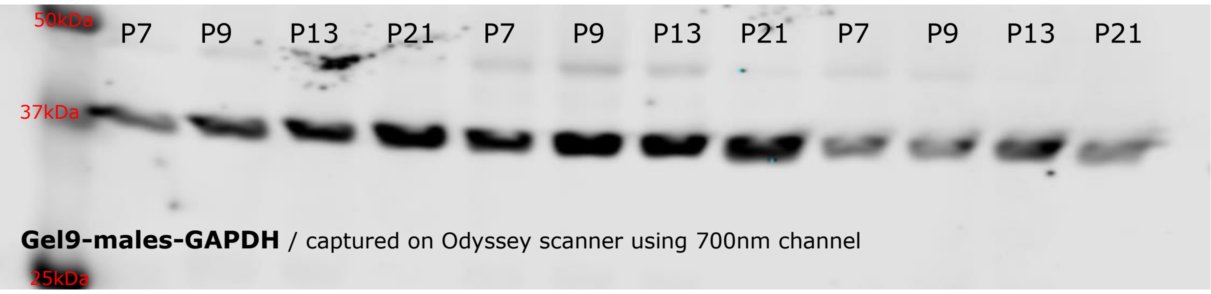

Gel 9	P7	P9	P13	P21	P7	P9	P13	P21	P7	P9	P13	P21
**GAPDH signal**	390000	546000	581000	885000	602000	1000000	826000	959000	348000	337000	575000	368000
**GIPR signal**	172000	160000	167000	120000	308000	377000	104000	146000	211000	105000	86800	48400
**GIPR/GAPDH**	0,441026	0,29304	0,287435	0,135593	0,511628	0,377	0,125908	0,152242	0,606322	0,311573	0,150957	0,131522

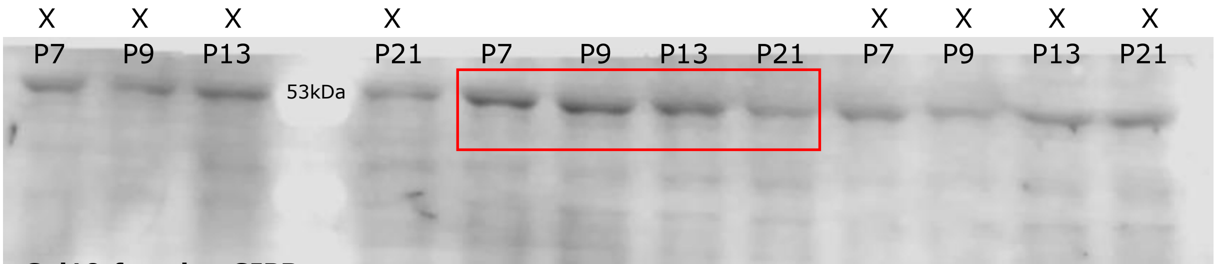

Gel10-females-GIPR / captured on Odyssey scanner using 800nm channel

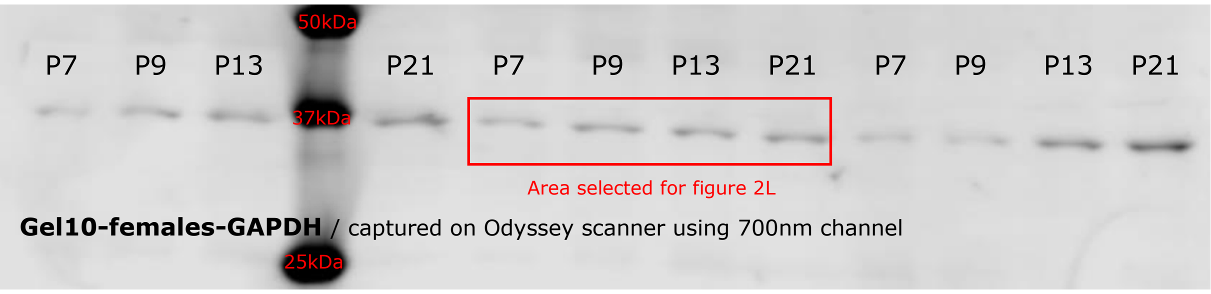

Gel10-females-GAPDH / captured on Odyssey scanner using 700nm channel

**Gel 10**	P7	P9	P13	P21	P7	P9	P13	P21	P7	P9	P13	P21
**GAPDH signal**	28800	48500	24200	99900	35200	51400	58500	57400	24100	27800	74500	13300<br>0
**GIPR signal**	61700	56700	75200	74300	12500<br>0	11700<br>0	90900	52300	71600	47200	72400	73600
**GIPR/GAPDH**	2,1423<br>61	1,1690<br>72	3,1074<br>38	0,743743<br>744	3,5511<br>36	2,2762<br>65	1,5538<br>46	0,911<br>15	2,9709<br>54	1,6978<br>42	0,9718<br>12	0,5533<br>83

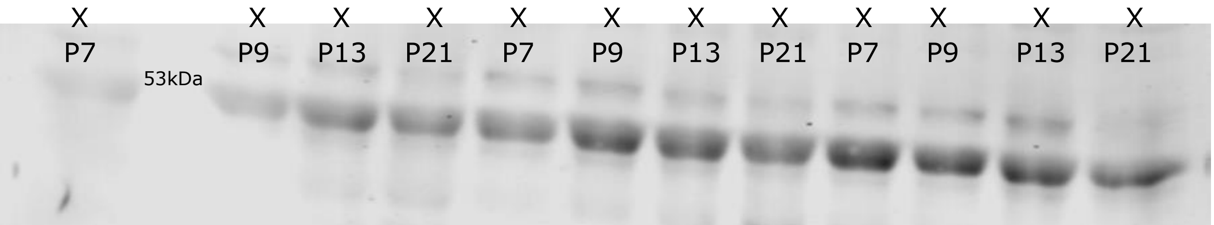

Gel11-females-GIPR / captured on Odyssey scanner using 800nm channel

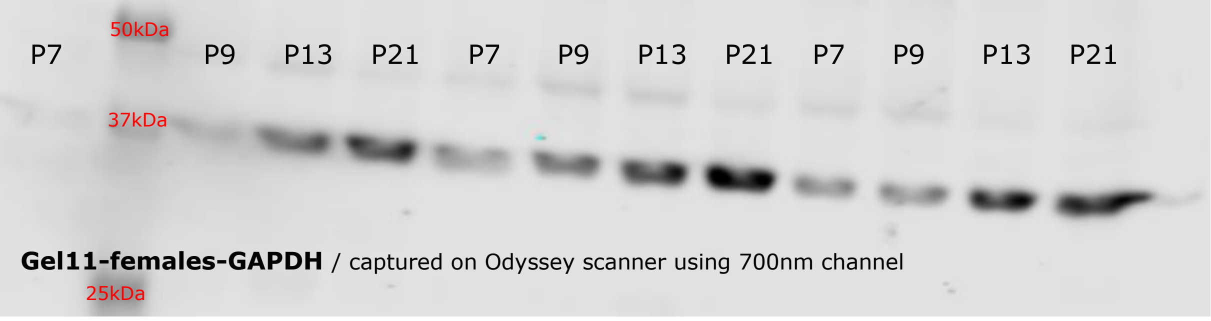

Gel11-females-GAPDH / captured on Odyssey scanner using 700nm channel

**Gel 11**	P7	P9	P13	P21	P7	P9	P13	P21	P7	P9	P13	P21
**GAPDH signal**	38500	205000	613000	823000	353000	466000	733000	103000<br>0	293000	29700<br>0	667000	871000
**GIPR signal**	57700	106000	226000	206000	230000	329000	302000	251000	359000	30700<br>0	331000	270000
**GIPR/GAPDH**	1,4987<br>01	0,5170<br>73	0,3686<br>79	0,2503<br>04	0,6515<br>58	0,7060<br>09	0,4120<br>05	0,2436<br>89	1,2252<br>56	1,033<br>67	0,4962<br>52	0,3099<br>89

CCKBR

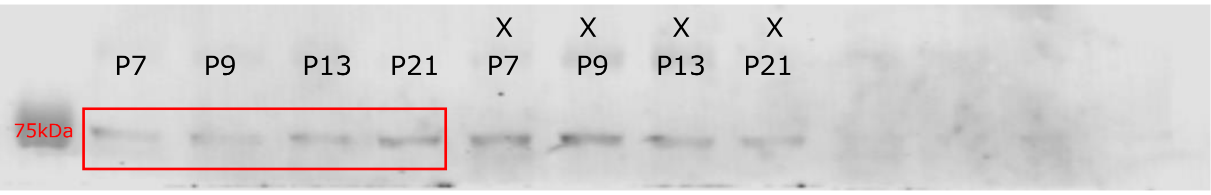

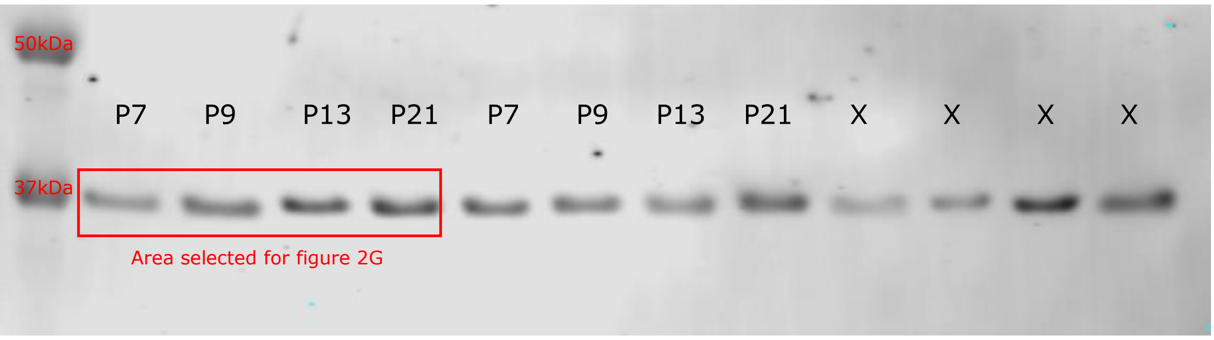

Gel 12	P7	P9	P13	P21	P7	P9	P13	P21
GAPDH signal	420000	676000	653000	756000	553000	550000	537000	448000
CCK2R signal	18900	16700	16700	24200	32800	40300	26700	16800
CCK2R/GAPDH	0.045	0.0247	0.0256	0.032	0.0593128	0.0733	0.0497	0.0375

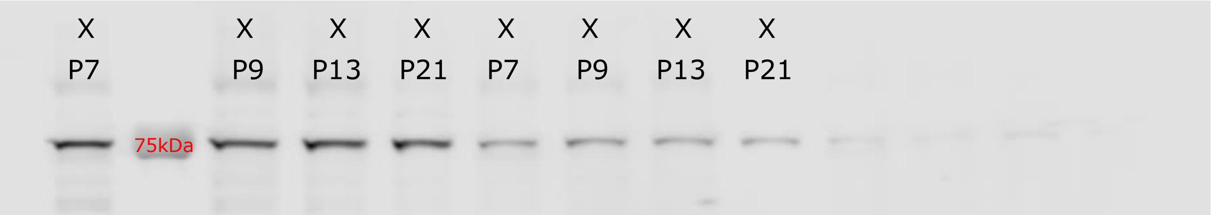

Gel13-males-CCK2R / captured on Odyssey scanner using 700nm channel

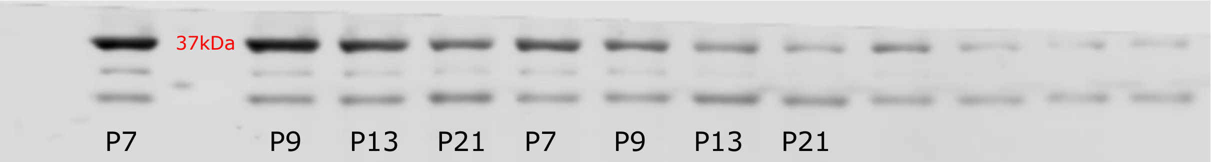

Gel13-males-GAPDH / captured on Odyssey scanner using 700nm channel

Gel 12	P7	P9	P13	P21	P7	P9	P13	P21
GAPDH signal	1280000	1410000	995000	613000	787000	571000	367000	200000
CCK2R signal	567000	556000	584000	508000	208000	230000	185000	127000
CCK2R/GAPDH	0,442969	0,394326	0,586935	0,828711256	0,264295	0,402802	0,504087	0,635

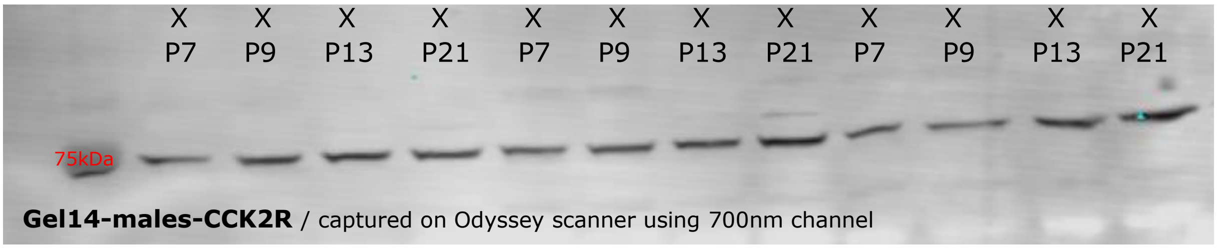

Gel14-males-CCK2R / captured on Odyssey scanner using 700nm channel

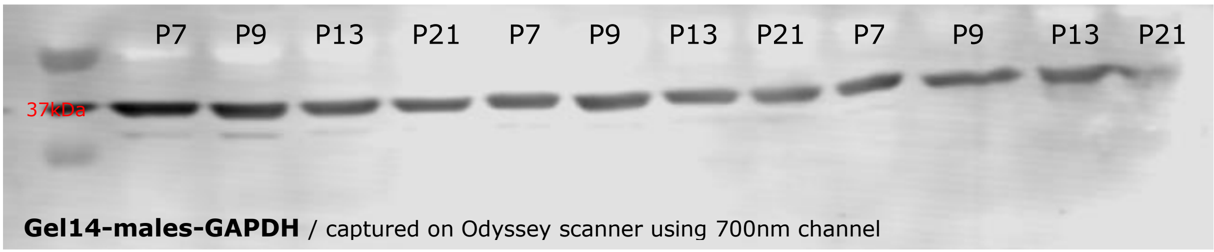

Gel14-males-GAPDH / captured on Odyssey scanner using 700nm channel

**Gel 14**	P7	P9	P13	P21	P7	P9	P13	P21	P7	P9	P13	P21
**GAPDH signal**	1840000	2010000	1350000	1440000	1550000	1680000	1160000	1180000	1290000	1330000	1460000	398000
**CCK2R signal**	566000	896000	970000	900000	815000	859000	834000	1050000	590000	555000	847000	-
**CCK2R/GAPDH**	0,307609	0,445771	0,718519	0,625	0,525806	0,51131	0,718966	0,889831	0,457364	0,417293	0,580137	-

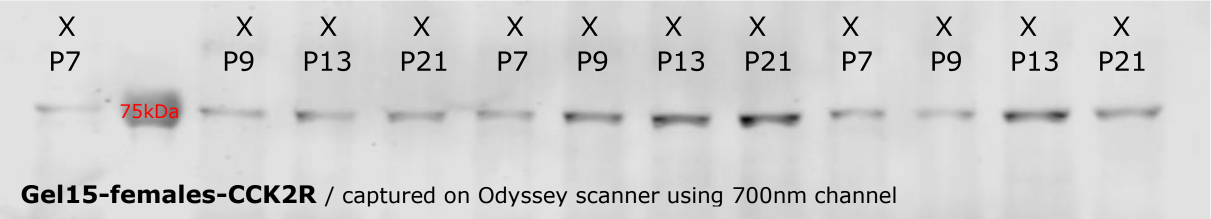

Gel15-females-CCK2R / captured on Odyssey scanner using 700nm channel

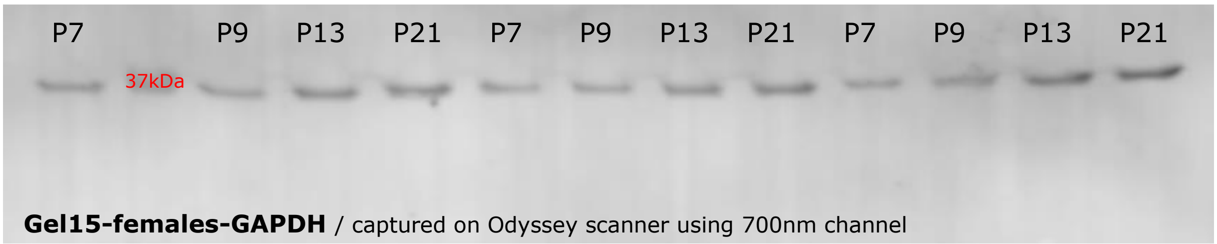

Gel15-females-GAPDH / captured on Odyssey scanner using 700nm channel

**Gel 15**	P7	P9	P13	P21	P7	P9	P13	P21	P7	P9	P13	P21
**GAPDH signal**	546000	430000	639000	716000	429000	374000	579000	663000	367000	542000	833000	880000
**CCK2R signal**	40800	58100	77100	72500	53000	117000	160000	190000	62700	45400	176000	99900
**CCK2R/GAPDH**	0,074725	0,135116	0,120657	0,101257	0,123543	0,312834	0,276339	0,286576	0,170845	0,083764	0,211285	0,113523

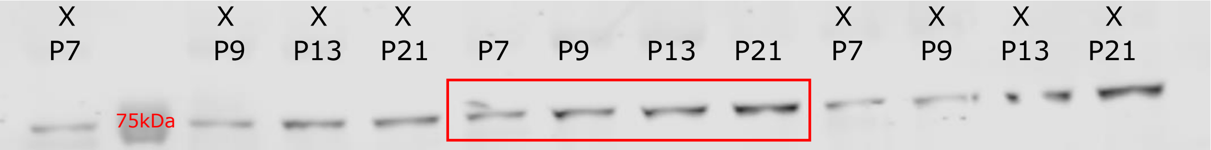

Gel16-females-CCK2R / captured on Odyssey scanner using 700nm channel

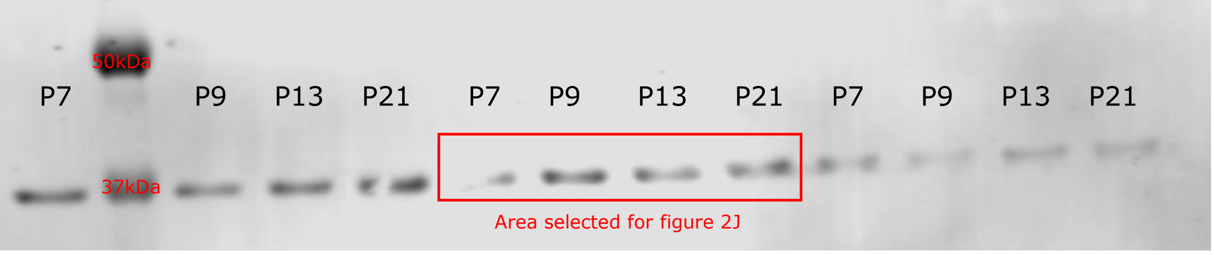

Gel16-females-GAPDH / captured on Odyssey scanner using 700nm channel

**Gel 16**	P7	P9	P13	P21	P7	P9	P13	P21	P7	P9	P13	P21
**GAPDH signal**	505000	341000	422000	568000	182000	575000	400000	408000	223000	223000	200000	144000
**CCK2R signal**	77700	68000	133000	135000	100000	151000	173000	236000	73700	68800	134000	258000
**CCK2R/GAPDH**	0,153861	0,199413	0,315166	0,237676056	0,549451	0,262609	0,4325	0,578431	0,330493	0,30852	0,67	1,791667
