# Supplementary material for: Gut-derived peptide hormone receptor expression in the developing mouse hypothalamus
Source: PLoS One. 2023 Aug 17;18(8):e0290043. doi: 10.1371/journal.pone.0290043 (PMC10434938; doi:10.1371/journal.pone.0290043)
Supplement: Attachment — Submitted filename: Cantacorps_Response to Reviewers.docx [file pone.0290043.s005.docx]

Dear Reviewer,

First and foremost, thank you for taking the time to review our manuscript. We understand that everyone is under pressure in today’s day in science. Given that reviewing is still a necessary part of the publication process, we are grateful that you accepted the request to review and took the time to provide valuable feedback and points of consideration. We feel we were able to address many of your points and have added additional data/experiments that we hope make a more comprehensive manuscript that can be considered for publication. You will find point by point answers to your comments below:

*5. Review Comments to the Author

Please use the space provided to explain your answers to the questions above. You may also include additional comments for the author, including concerns about dual publication, research ethics, or publication ethics. (Please upload your review as an attachment if it exceeds 20,000 characters)*

*Reviewer #1: Cantacorps et al have examined gut-derived peptide receptor expression in the hypothalamus, at the gene and protein level, across the early post-natal period in both sexes. These results are of interest as the in utero environment can impact on hypothalamic development and lead to later loss of metabolic homeostasis and the development of obesity, but the mechanisms are unknown. In order to understand how hypothalamic development is altered, studies such as this one examining developmental processes under normal circumstances are first required. The introduction is clear and explains the relevance and importance of the study, and the methods are detailed and easy to follow. The discussion places the new results in the context of what is already known in this field, but would benefit for some text discussing the limitations of the findings in this study. Although the data is novel, overall the manuscript does not contain a lot of data. I have some other queries and concerns as detailed below:*

First, that you for the perfect summary of the manuscript and further, the acknowledgment of the importance to study the role of these gut hormones in normal development before we can tackle understanding their function or change in signaling in response to early altered metabolic environments. We agree that that data is not extensive, which is why we have proposed it as a short report in another PLOS journal, and it was transferred here, where Research Article is the only option. Further, the collection of these timepoints across development was a laborious effort, and the initial results are interesting to the field and we deemed it better to submit as is, since we do not have the capacity (nor the funding) to pursue it further at the moment. We hope that the hypothesis tested, the results we have prepared for submission and the discussion this can contribute to the field will support accepting this manuscript for publication. We were able to include more data in the revision that support the initial studies and expand the understanding of the developmental role of these gut-derived peptides signaling aspects and we hope with the addition of these points the improved manuscript can be accepted for publication.

*- In Figure 1B and 1C there appears to be some clustering within timepoints of the body weight and glucose data. Is this related to litter (i.e. littermates sharing a body weight similar to each other, but not other animals?). Related to this point, the expression levels of the receptors at gene expression level is very varied within each time point, creating large error bars on the graphs. Is any of this explained by litter differences or is it correlated to body weight/ glucose?*

We did not detect any differences on body weight within each time-point related to litter, as it can be seen in the graphs (from Figure 1) here provided for body weight color-coded by litter, where it’s shown that single data points belonging to littermates are not clustered together.

Notwithstanding, for the glucose levels some of the data points belonging to same litter appear to be clustered together (i.e. P7 males, P9 females or P21 males). This could be due to having had access to food recently or not for that litter. Still, we did not find any significant correlation between protein expression of the receptors and glucose levels, whereas we did obtain significant correlations for all the receptors with body weight. The correlation graphs to glucose levels and body weight are now included as Figure 3, this data is also included below for reference.

Interestingly both InsR and GIPR are negatively correlated with body weight, and could indicate the early role of these receptors in the brain for neuronal outgrowth. Indeed GIP-GIPR activation was recently shown to affect polarization and growth of postnatal cortical neurons [45, 46].

*- A limitation of this study is the authors have used whole hypothalamus which may mask changes happening in individual nuclei (relevant as these receptors are not expressed in all hypothalamic nuclei). I realize this will not be possible to correct post- experiment but do the authors have any pilot data on different nuclei? This limitation should be noted in the discussion.*

We agree that this is a limitation of the study, however, dissection of the hypothalamus itself is extremely difficult in such small offspring brain, and in order to generate enough tissue from each individual subregion to analyze these results, a significantly higher number of animals would need to be sacrificed. In order to first determine if there are in fact any changes on the receptor expression generally in the whole hypothalamus across development, we opted to study the whole region first. However, in order to simplify the result comparison between mRNA and protein, we have updated the figure to reflect the same timepoints in both studies so direct comparisons can be made. Further, statistical outliers were determined using the delta CT values, prior to calculation of the 2^-ddCT. Given our results, a further subnuclei expression profile across development would be an interesting next step, but is not feasible in the span of this manuscript. However, we do agree that is important to understand the complex spatial distribution of these receptors through the hypothalamus, and with the reviewers’ suggestion, we were able to include data from adult hypothalamic subnuclei, where we were able to dissect individual brain regions and perform qPCR analysis of the different peptide hormone receptors in the adult female brain. We have now included this data as Supplemental Figure 1 of the manuscript and added the following text to the results section and subsequent discussion as well as the information about the animals etc to the corresponding methods section. Furthermore, we generated a figure with in situ hybridization data from the Allen Developing Mouse Brain Atlas in order to discuss the spatial distribution expression of the receptors during the early developmental phase and have included this as Figure 4 in the manuscript.

Results:

<<2.6 Distribution of gene expression in the hypothalamus.

In order to determine the likely most effected subregions of the hypothalamus due to the alterations across development, we targeted tissue collected to the individual hypothalamic subnuclei, including the ARC, VMH/DMH, PVH and LH from adult animals (Supplementary Fig. 1). In adult mice, the distribution of the *Insr* expression is relatively evenly distributed throughout the hypothalamus, with the highest concentration of gene expression in the ARC and VMH/DMH (Supp Fig. 1 A, [F(3, 21)=8.787; p<0.001]). *Cckbr* appears to be widely distributed throughout the hypothalamus in both our data [F(3, 22)=0.8842; p=n.s.] and in the in situ hybridization (ISH) data from Allen Brain Atlas (Supp. Fig. 1 B). *Glp1r* appears to be mainly expressed in the ARC, and to a lesser extent in the other subnuclei [F(3, 19)=6.127; p=0.004], which can also be seen on the ISH images from the Allen Brain Atlas (Supp Fig. 1 C). *Gipr* is predominantly found in the ARC with expression also being identified in the LH, the VMH/DMH and the PVH [F(3, 22)=4.778; p=0.010]. To further probe the spatial distribution of gene expression in the early developmental period (Figure 4), we aligned ISH data from the Allen Developing Mouse Brain Atlas to determine the expression correlates across development for the *Insr*, *Cckbr*, and *Glp1r* (*Gipr* data not available). Here interestingly, in contrast to the compartmentalized expression of *Cckbr* and *Glp1r*, the gene expression of the *Insr* appears to be evenly distributed throughout the hypothalamus and displays what appears to be a slight decrease between P14 and P28 (Fig. 4A) which supports the Western blot data showing this same decrease in both males and females. In contrast to the wide expression of *Insr*, it can clearly be seen that the *Cckbr* is highly expressed in the VMH by P14(Fig. 4B), despite being relatively dispersed in the hypothalamus of adult animals. Similarly, the *Glp1r* expression is highly compartmentalized into the PVH region in early development reaching its highest level by P14, while getting more widely distributed throughout the hypothalamic subnuclei with age (Fig. 4C). Unfortunately, the only available developing brain ISH data is in male animals, warranting a further understanding as to the distribution within the hypothalamus subregions in female animals and their expression changes across development. >>

Discussion:

<<The targeted analysis of the receptors’ gene expression within the hypothalamic subnuclei in the adult mouse brain combined with the ISH data available from the Allen Developing Mouse Brain Atlas allowed us to hypothesize which subregion in particular might be more affected by the protein expression changes here assessed during development. Potentially, the overall increased expression of CCK2R and GLP1R that we observed during early postnatal life could be compartmentalized into the VMH and the PVH, respectively. In contrast, the decline observed in both InsR and GIPR expression might be affecting the whole hypothalamus. Notwithstanding, a further analysis of the individual hypothalamic subnuclei expression profile across development would be necessary to corroborate this.>>

- *The authors have used the 2^-ΔΔCt comparative method to analyse qPCR results and state groups are normalised to the P3 timepoint. Is this within sex? (i.e. is it possible to compare between sexes to examine whether there are sex differences in mRNA quantity rather than just seeing the changes across a time course?). Also can the authors explain why Fig 2C the P3 time point is sitting at around 5 on the y axis- have the authors used a geomean?*

Upon reanalysis of the raw data, one data point in the P3 dataset was extremely different than the remaining samples in the group. However, using the approach above (determining statistical outliers based on the deltaCT value prior to moving forward with relative expression analysis) and further limiting the graph to matched timepoints to the Western Blot studies eliminate this issue.

In order to directly compare results between sexes, we reperformed this gene expression analysis limited to the timepoints matching the Western Blot data. In doing so, we could directly compare gene expression between sexes by including all samples on the same qPCR plate. Here we do not see a significant difference in gene expression between males and females for any of the genes assessed at either timepoint (Table 1). The data comparing gene expression between sexes is included below for reference to the reviewer and Figure 2 of the manuscript and the corresponding Results section has been updated with the new qPCR data.

| **Gene** | **Sex factor** | **Age factor** |
| --- | --- | --- |
| *Cckbr* | F (1, 36) = 0,3036; p=0.585 | F (3, 36) = 2,624; p=0.065 |
| *Glp1r* | F (1, 36) = 0,02878; p=0.866 | F (3, 36) = 0,8929; p=0.454 |
| *Gipr* | F (1, 36) = 0,8368; p=0.3664 | F (3, 36) = 2,579; p=0.0687 |

**Table 1.** Results of two-way ANOVA analysis.

- *There also seem to be several individual points in the gene expression graphs in fig 2 where gene expression is nearly at zero. Can the authors explain this- do they think the gene expression is actually zero in these animals or is it a problem with tissue dissection/ RNA extraction/ reverse transcription? The spread of the data in each timepoint in Fig 2A-F is very variable aim this is hindering interpretation of the data.*

We agree with the reviewer that the variability in these samples for the qPCR is quite high. However, we would like to stress that we are attempting qPCR from dissected hypothalamic tissue from early developmental stages. On top of that we are also assessing the expression of receptors which are inherently lowly expressed at the mRNA level even in the adult brain and all of the receptors are also regulated extensively at the protein level. Thus, while we analyzed the gene expression as a first potential indication of changes across development, we are of the opinion that the Western Blot data depicting the actual changes in protein expression are more pivotal for the discussion. When trying to assess receptor expression with qPCR there is always a risk of too low expression leading to inconclusive results, high Ct values and undetermined samples. In order to address this issue, we were able to extract RNA from additional samples and targeted only the 4 time points also addressed in the Western Blot data, by doing so we could also then analyze sex specific effects. The new results of this analysis can be found in the updated version of Figure 2.

Furthermore, we added the following to the discussions section:

“While we aimed to dissect a link between gene and protein expression of the individual GPCRs, our data indicate that there is not a strong correlation between the two measurements. Indeed, while there is argumentation that mRNA and protein levels are strongly correlated [34, 35], these results are largely based on the analysis of highly abundant genes represented in most sequencing analysis. However, GPCRs are among the most abundant genes which are not detected in traditional sequencing analysis of mRNA abundance [36]. Indeed, among the top 50 most highly abundantly expressed GPCRs the lower limit is approximately 5 transcripts per million reads [37], and the transcripts we are detecting in this sample set are significantly lower in their expression, and thus more highly variable in their detection as well. GPCRs have evolved to have very complex mechanisms of regulation, through desensitization, internalization and recycling, warranting their protein expression to be the more determinant factor in their role in development [38, 39]. Therefore, the findings of protein expression across early periods of life more strongly support a role of these proteins in mediating aspects of growth and development. Further studies will be necessary to uncover potential changes to their membrane expression and activity regulation in early development.”

*- All of the genes examined seem to be at their lowest (in both males and females) around the P9-P13 timepoint. Can the authors confirm that this is not due to a technical error (where all the mice collected on the same day, or processed by a different researcher?). If this possibility is ruled out, have the authors considered an effect of the leptin surge which is occurring at this time point- do they have data on serum leptin level in the same mice to plot a correlation?*

In response to the comment about potential causes of variability, we critically reviewed the person performing the hypothalamic dissections in all studies. Coincidentally in the qPCR related studies, all dissections were performed by a single researcher, and post processing (RNA Extraction, cDNA synthesis) were distributed between the researcher and a technical assistant, but no pattern was evident that would suggest a technical error resulting in the effects we see in the data. Due to the differing ages for each timepoint, the samples were collected on different days, but as the timepoints P9-P13 represent samples from more than 1 litter, we do not see an overall effect of day of collection on distribution of data.

We agree that gene expression across these timepoints however we would also point out that in terms of protein expression the P9-P13 timepoint shows an increase in INSR and GLP1R, and a decrease in CCK2R and GIPR. While we were not able to collect serum from the samples used for qPCR, we were able to analyze the leptin plasma levels of samples from the same animals used for Western blot data. As the postnatal leptin surge affects the growth of the animals in this time period, we applied the reviewer’s suggestion and plotted the protein expression versus circulating leptin levels to determine if there might be a link, either directly or indirectly, on the levels of these hormone receptors. This data has now been added as a new Figure 5 to the manuscript and into the Results section. We appreciate the suggestion from the review to assess this potential link.

The following texts were also added to the manuscript:

Methods section:

“4.8 ELISA

Trunk blood samples from pups aged 7, 9, 13 and 21 days old were collected in EDTA-tubes (Sarstedt; Cat#16,444), centrifuged at 2,500 g for 25 min at 4°C and plasma aliquots were stored at -80°C. Circulating leptin levels were quantified using the Mouse leptin ELISA kit (Crystal Chem; Cat# 90030, Lot# 21OCML444) following the manufacturer’s instructions and absorbance was measured at 450/630 nm using a microplate reader (BMG labtech).”

Results section:

“2.7 Protein expression changes across development and role of the postnatal leptin surge.

In the early postnatal period it is known that particular hormonal surges are linked to postnatal growth and development, also within the brain. The hallmark of this is the postnatal leptin surge [32]. To determine a potential meditating effect of the postnatal leptin surge on expression of other key gut-derived peptide hormone receptors, we analyzed leptin levels in plasma collected from mice at P7, P9, P13, and P21. In both male and female animals, no correlation was noted between circulating leptin levels and protein expression of InsR (r2=0.0000345, p=0.9750), CCK2R (r2=0.03645, p=0.3607), GLP1R (r2=0.00155, p=0.8392) or GIPR (r2=0.001515, p=0.8297) (Figure 5A-D). Thus, suggesting that inherent changes to leptin, known to affect growth and development of neuronal circuits in this early developmental time period, does not directly impact the expression of other gut-derived peptide hormone receptors.”

*- There really is not much synergy between the gene and protein expression across the data. This could of course be due to post-transcriptional mechanisms, but this should be addressed in the discussion.*

The following section was added to the discussion to reiterate the potential for mRNA and protein expression levels of GPCR to not be directly correlated.

“While we aimed to dissect a link between gene and protein expression of the individual GPCRs, our data indicate that there is not a strong correlation between the two measurements. Indeed, while there is argumentation that mRNA and protein levels are strongly correlated [34, 35], these results are largely based on the analysis of highly abundant genes represented in most sequencing analysis. However, GPCRs are among the most abundant genes which are not detected in traditional sequencing analysis of mRNA abundance [36]. Indeed, among the top 50 most highly abundantly expressed GPCRs the lower limit is approximately 5 transcripts per million reads [37], and the transcripts we are detecting in this sample set are significantly lower in their expression, and thus more highly variable in their detection as well. GPCRs have evolved to have very complex mechanisms of regulation, through desensitization, internalization and recycling, warranting their protein expression to be the more determinant factor in their role in development [38, 39]. Therefore, the findings of protein expression across early periods of life more strongly support a role of these proteins in mediating aspects of growth and development. Further studies will be necessary to uncover potential changes to their membrane expression and activity regulation in early development.”

**References**

32.	Ahima, R.S., D. Prabakaran, and J.S. Flier, Postnatal leptin surge and regulation of circadian rhythm of leptin by feeding. Implications for energy homeostasis and neuroendocrine function. J Clin Invest, 1998. 101(5): p. 1020-7.

34.	Buccitelli, C. and M. Selbach, mRNAs, proteins and the emerging principles of gene expression control. Nat Rev Genet, 2020. 21(10): p. 630-644.

35.	Wilhelm, M., et al., Mass-spectrometry-based draft of the human proteome. Nature, 2014. 509(7502): p. 582-7.

36.	Fagerberg, L., et al., Analysis of the human tissue-specific expression by genome-wide integration of transcriptomics and antibody-based proteomics. Mol Cell Proteomics, 2014. 13(2): p. 397-406.

37.	Sriram, K., et al., Detection of GPCR mRNA Expression in Primary Cells Via qPCR, Microarrays, and RNA-Sequencing. Methods Mol Biol, 2021. 2268: p. 21-42.

38.	Gurevich, V.V. and E.V. Gurevich, GPCR Signaling Regulation: The Role of GRKs and Arrestins. Front Pharmacol, 2019. 10: p. 125.

39.	Magalhaes, A.C., H. Dunn, and S.S. Ferguson, Regulation of GPCR activity, trafficking and localization by GPCR-interacting proteins. Br J Pharmacol, 2012. 165(6): p. 1717-1736.

45.	Teng, L., et al., GIP-GIPR promotes neurite outgrowth of cortical neurons in Akt dependent manner. Biochem Biophys Res Commun, 2021. 534: p. 121-127.

46.	Okawa, T., et al., Sensory and motor physiological functions are impaired in gastric inhibitory polypeptide receptor-deficient mice. J Diabetes Investig, 2014. 5(1): p. 31-7.
